# Data Extraction From Oncology Imaging Reports by Large Language Models: A Comparative Accuracy Study

**DOI:** 10.1200/CCI-26-00002

**Published:** 2026-08-10

**Authors:** Lea P. Passweg, Johannes M. Schwenke, Christof M. Schönenberger, Flavio Locher, Julia Picker, Manuel Dieterle, Benjamin Thiele, Dimitri Hasler, Alessia Danelli, Andreas M. Schmitt, Tobias Heye, Thomas Stojanov, Matthias Briel, Benjamin Kasenda

**Affiliations:** ^1^ Division of Medical Oncology, University and University Hospital Basel, Basel, Switzerland; ^2^ Division of Clinical Epidemiology, Department of Clinical Research, CLEAR Methods Center, University and University Hospital Basel, Basel, Switzerland; ^3^ Medical University Clinic, Kantonsspital Aarau (KSA), Aarau, Switzerland; ^4^ Division of Digitalization & ICT, University Hospital Basel, Basel, Switzerland; ^5^ Clinic of Radiology and Nuclear Medicine, University and University Hospital Basel, Basel, Switzerland; ^6^ Surgical Outcome Research Center, University and University Hospital Basel, Basel, Switzerland

## Abstract

**PURPOSE:**

Manual data extraction from clinical text is resource-intensive. Locally hosted large language models (LLMs) may offer a privacy-preserving solution, but their performance on non-English data remains unclear. We investigated whether the accuracy of locally hosted LLMs is noninferior to human accuracy when determining metastasis status and treatment response from German radiology reports.

**METHODS:**

In this retrospective comparative accuracy study, five locally hosted LLMs (llama3.3:70b, mistral-small:24b, qwq:32b, qwen3:32b, and gpt-oss:120b) were compared against humans. A ground truth was established via duplicate human extraction and adjudication of discrepancies by a senior oncologist. The study was conducted at a tertiary referral hospital in Switzerland. We randomly sampled 400 radiology reports from adult patients with cancer (computed tomography, magnetic resonance imaging, positron emission tomography) generated between January 2023 and May 2025 and split them into a prompt optimization set (n = 100) and test set (n = 300). Primary outcomes were noninferiority (5 percentage points [pp] margin) of LLM classification accuracy compared with human accuracy for metastasis status (presence/absence by anatomic site) and treatment response categories. Secondary outcomes included accuracy for primary tumor diagnosis and radiologic absence of tumor.

**RESULTS:**

The analysis included 400 reports from 317 patients. In the test set (n = 300), the human accuracy for metastasis status was 98.4% (95% CI, 98.0 to 98.8). All LLMs were noninferior; gpt-oss:120b performed best (97.6% accuracy; difference, –0.8 pp [90% CI, –1.3 to –0.3 pp]). For response to treatment, the human accuracy was 86.0% (95% CI, 83.2 to 88.8). All LLMs were inferior; the most accurate model, gpt-oss:120b, achieved 78.3% (difference, –7.7 pp [90% CI, –11.6 to –3.8 pp]).

**CONCLUSION:**

In this study, LLMs were noninferior to human accuracy for classification of metastasis status but were inferior for response to treatment assessment.

## INTRODUCTION

Structured clinical data are essential for quantitative research, including descriptive statistics, prediction modeling, and clinical trials. Valuable clinical information, such as information on treatment response in patients with cancer, is typically embedded in unstructured free-text clinical notes in electronic health records, such as radiology or consultation reports. Manual extraction of these data is resource-intensive and error-prone and depends on training of data coders.^1,2^ This challenge is particularly critical in oncologic studies, where radiologic treatment response evaluation frequently serves as a primary outcome.

**CONTEXT**

**Key Objective**
Can locally hosted large language models (LLMs) match human performance when extracting sites of metastases and response to treatment from radiology reports of patients with cancer?
**Knowledge Generated**
In this preregistered, single-center study of 300 German radiology reports, all evaluated LLMs were noninferior to humans in extracting the presence or absence of metastasis by organ site, but were inferior to humans in classification of response to treatment. The findings support the use of locally hosted LLMs for explicit extraction tasks.
**Relevance *(J.L. Warner)***
This study has two important findings: (1) LLMs trained presumably on primarily English texts are portable to non-English medical applications and (2) clinical tasks must be selected carefully, as shown in the mixed performance with LLM non-inferiority for metastatic classification but consistent inferiority for treatment response assessment.*
*Relevance section written by *JCO Clinical Cancer Informatics* Editor-in-Chief Jeremy Lyle Warner, MD, MS, FAMIA, FASCO.

Recent advances in large language models (LLMs) have shown potential in data extraction and classification.^3-5^ Locally hosted LLMs preserve privacy and security compared with closed-source alternatives as sensitive patient data remain within a health service provider's secure IT infrastructure.^6^ It is unclear how well locally hosted LLMs perform on real-world, particularly non-English, radiology reports of patients with cancer and whether their outputs are reliable enough to support research and clinical workflows.

We tested a secure, LLM-based, automated pipeline which extracts information on primary tumor diagnosis, metastasis location, and response to treatment of patients with cancer from radiology reports. To assess the accuracy of our automated pipeline, we compared data extraction by humans with data extracted using our pipeline.

## METHODS

### Study Design and Setting

This retrospective study was conducted at the University Hospital Basel, Switzerland. All data processing occurred within the secure hospital network. Extracted data were stored in a locally hosted REDCap database.^7^ This study is reported in accordance with the TRIPOD-LLM reporting guidelines.^8^

### Radiology Reports

German radiology reports were retrieved from the Clinical Data Warehouse (CDWH) of the hospital; the data flow is shown in the Data Supplement (Data Supplementary, Fig S1). We included reports from computed tomography (CT), magnetic resonance imaging (MRI), and positron emission tomography-CT (PET-CT) ordered by physicians of the Division of Medical Oncology between January 2023 and May 2025, in adult (18 years and older) patients with cancer who had provided the hospital's General Research Consent.^9^ To allow meaningful response assessment and minimize reports on baseline-staging, examinations had to occur ≥2 months after the first outpatient oncology appointment. Reports clearly irrelevant for tumor response assessment according to their CDWH identifier, such as reports on imaging-guided interventions, were excluded. Based on a power simulation, we randomly sampled 400 imaging reports and split the sample into a prompt optimization set (n = 100) and test set (n = 300).

### Extraction Tasks

The data extraction comprised four tasks: (1) classification of primary tumor diagnosis from the medical history embedded in the radiology report as one of 28 prespecified SNOMED Clinical Terms categories (eg, colorectal cancer); (2) classification of metastasis status, as present or absent for each metastasis region (eg, lungs, liver, bone, brain, lymph nodes), depending on whether the region of interest was relevant for the respective imaging examination; and (3) classification of treatment response (on PET-CT as complete response, partial response, stable disease, progressive disease, or not applicable; on CT/MRI as response, stable disease, progressive disease, not applicable). The “not applicable” category was assigned when the imaging study was not performed for treatment monitoring (eg, baseline staging). (4) classification of radiologic absence of any tumor (yes/no). We a priori decided against an attempt to differentiate between complete response and partial response of reports from CT or MRI, based on clinical experience that this distinction can be challenging. We did not categorize responses according to the RECIST or the Response Assessment in Neuro-Oncology criteria because these are not routinely applied and reported outside of clinical trials.

### Ground Truth

Two groups of human coders (medical master students and medical doctors) independently extracted data in duplicate, using the same written guidelines (see Supplementary Methods). When the two coders agreed, their answer became the ground truth. If there was disagreement, this was adjudicated by a senior oncologist, establishing the ground truth. Inter-rater agreement between the two human coders and each human coder and each LLM was assessed using raw percent agreement and Cohen's kappa (κ).^10^ To assess the robustness of this human-derived ground truth, we performed a post hoc sensitivity analysis on the test set. We identified all instances where the best-performing model disagreed with the ground truth for the primary outcomes. These discrepancies were reviewed by two senior oncologists (B.K., A.M.S.) who were blinded to the source of the extraction. This review established a revised ground truth used specifically for the sensitivity analysis.

### LLMs and Prompting

We evaluated five locally hosted LLMs using the hospital's graphics processing unit (GPU) cluster. The cluster consists of a high-density GPU server equipped with dual 48-core x86 processors, approximately 2.3 TB of DDR5 system memory, and eight NVIDIA H200 PCIe Gen5 GPUs, each providing 141 GB of high-bandwidth memory. Models were locally deployed using Ollama version 0.12.2 and accessed through R using ellmer version 0.3.0.^11,12^ We evaluated the performance of the following models: llama3.3:70b (full name: llama3.3:70b-Instruct-q5_K_M, knowledge cutoff December 2023, release date December 6, 2024),^13^ mistral-small:24b (full name: mistral-small:24b-Instruct-2501-q4_K_M, knowledge cutoff October 2023, release date January 30, 2025),^14^ qwq:32b (knowledge cutoff November 2024, release date March 5, 2025), qwen3:32b (knowledge cutoff not stated, release date April 29, 2025),^15^ and gpt-oss:120b (knowledge cutoff September 2024, release date August 5, 2025).^16^ We selected open-source models in the 24B-120B parameter range based on local infrastructure constraints. Substantially larger models were not compatible with local deployment in our setting. Models were used without additional fine-tuning and prompted in English as this has been shown to be optimal for classification of non-English text.^17-19^

Prompts were iteratively refined through trial and error on the prompt optimization set (n = 100) and applied to the test set (n = 300). We implemented a two-step pipeline where models provided free-text reasoning for generating structured JSON. This led to improvements in performance for the extraction of tasks across all models except gpt-oss:120b which required direct output (Data Supplement, Fig S2, Supplementary Methods). Prompts were adapted to the imaging examination (PET-CT *v* CT/MRI; body region of the imaging examination; Data Supplement, Figs S3 and S4). Inference settings were fixed across models with a context window of 10,000 tokens and a temperature of 0 to maximize reproducibility. For the exact prompts and implementation details, see Supplementary Methods and code on GitHub.^20^

### Outcomes

The two prespecified primary outcomes were the difference in human versus LLM accuracy for classification of metastasis status and treatment response assessment.^21^ Secondary outcomes were the accuracy for classification of the primary diagnosis, radiologic absence of tumor, and overall accuracy across all four tasks, as well as the mean time of extraction per report. Exploratory analyses included sensitivity, specificity, and negative and positive predictive values (PPVs).

### Statistical Analysis

For each outcome, the initial human extractions and responses from each LLM were compared against the established ground truth. To compare the performance of LLMs with that of humans, we used logistic regression models to estimate quantities of interest. We accounted for within-report correlation using cluster-robust standard errors at the report level.^22,23^ We tested for noninferiority in the primary outcomes against a prespecified margin of -0.05 for the difference in accuracy. This margin served as a pragmatic feasibility threshold to determine if model performance warranted further development, rather than a strict clinical safety limit. Standard errors were estimated using the delta method.^24^ For exploratory outcomes (sensitivity, specificity, negative predictive value [NPV], PPV, F1 score), we used bootstrapping, with 1,000 repetitions, as the delta method estimates produced confidence intervals crossing 0 or 1. We did not adjust for multiplicity across LLMs and tasks. To check the robustness of our findings, we performed a Bayesian sensitivity analysis with weakly informative priors.

We estimated the human time per report using REDCap audit logs, which timestamped when a data extraction form was saved. For each user, we calculated the time gap (in seconds) between consecutive saves. Gaps exceeding 300 seconds were classified as breaks and were treated as missing values. These missing values were then imputed using the mean of all observed gaps across the data set. Mean gap time served as the approximate human time per report. For the LLMs, we recorded the total processing time for all reports and calculated the mean time per report. All analyses were performed in R (version 4.4.3) using marginaleffects (version 0.30.0) for model-based predictions and the tidyverse (version 2.0.0) for data handling and visualization.^25-27^

### Sample Size Rationale

We conducted a power simulation.^20^ For metastasis classification, with a conservative estimate of three metastasis locations to classify as absent or present per report, we estimated power is to be >95% to detect noninferiority at an α of 0.05, if the true LLM accuracy were to equal human accuracy. For classification of treatment response assessment, our estimated power is 70%-90%, depending on report heterogeneity. Overall, 300 reports provide high power for metastasis classification and adequate power for the response to treatment.

### Ethics Approval and Consent to Participate

Ethical approval was obtained from the Ethics Committee of Northwestern Switzerland (BASEC 2025-01036). The study design and analyses were preregistered on OSF.^21^

## RESULTS

### Sample Characteristics

We included a total of 400 radiology reports from 317 patients (mean age 63 ± 15 years; 32% female). Most reports were CT or MRI (89%, n = 355), with the remaining 11% (n = 45) being PET-CT scans (Table 1).

**TABLE 1.** Patient Characteristics, Imaging Modality, Primary Diagnosis, Metastasis Location, Treatment Response on CT/MRI and PET-CT, and Tumor-Free Status of the Study Cohort (N = 400)

Patient Characteristic (N = 317)	Imaging Report Characteristics (N = 400), %
Age, years, mean (SD)	63.28 (15.1)
Female sex	103 (32)
Imaging modality	
PET–CT	45 (11)
CT/MRI	355 (89)
Primary diagnosis	
Lung tumor (including mesothelioma)	95 (24)
GI tumors	63 (16)
Primary brain tumor	56 (14)
Primary skin tumor (including melanoma and non-melanoma)	41 (10)
Other^a^	145 (36)
Metastasis location	
Lymph nodes	132 (33)
Bone	108 (27)
Lungs	96 (24)
Liver	68 (17)
Other^b^	108 (27)
Treatment response (CT/MRI; n = 355)	
Response	174 (49)
Stable disease	80 (23)
Progression	73 (21)
Not applicable	28 (8)
Treatment response (PET–CT; n = 45)	
Complete response	19 (42)
Partial response	11 (24)
Progression	11 (24)
Not applicable	3 (7)
Stable disease	1 (2)
Tumor-free status	
No	244 (61)
Yes	125 (31)
Not applicable	31 (8)

Abbreviations: CT, computed tomography; MRI, magnetic resonance imaging; PET–CT, positron emission tomography–computed tomography; SD, standard deviation.

a Other primary tumors comprise lymphoma: 33 (8%), prostate cancer: 26 (7%), sarcoma: 23 (6%); head and neck tumors: 18 (5%), cancer of the urinary tract (including ureter and bladder): 13 (3%), others; eight (2%), testicular cancer: eight (2%), no malignant disease, six (2%); kidney tumor: five (1%), unclear: two (1%), breast cancer: one (<1%), cancer of unknown primary: one (<1%), thyroid cancer: one (<1%).

b Other metastasis locations comprise soft tissue: 26 (7%), CNS: 20 (5%), pleura: 18 (5%), peritoneum: 14 (4%), adrenal: 12 (3%), pancreas: eight (2%), spleen: six (2%), and kidney: four (1%).

### Inter-Rater Agreement

Across the 400 radiology reports, the two human coders independently classified a total of 4,669 data items in duplicate. This comprised 3,469 assessments of specific metastasis sites and 400 classifications, each for primary diagnosis, treatment response, and radiologic tumor-free status (no evidence of disease). Agreement was 91.5% for primary diagnosis (κ = 0.90), 96.6% for metastasis status (κ = 0.77), 80.5% for radiologic tumor-free status (κ = 0.62), and 76.2% for response to treatment (κ = 0.68). Agreement between each model and the human coders was lower, except for gpt-oss:120b, which showed a level of agreement with each human coder similar to that observed among the human coders themselves (Data Supplement, Table S1).

### Metastasis Status

**FIG 1.**
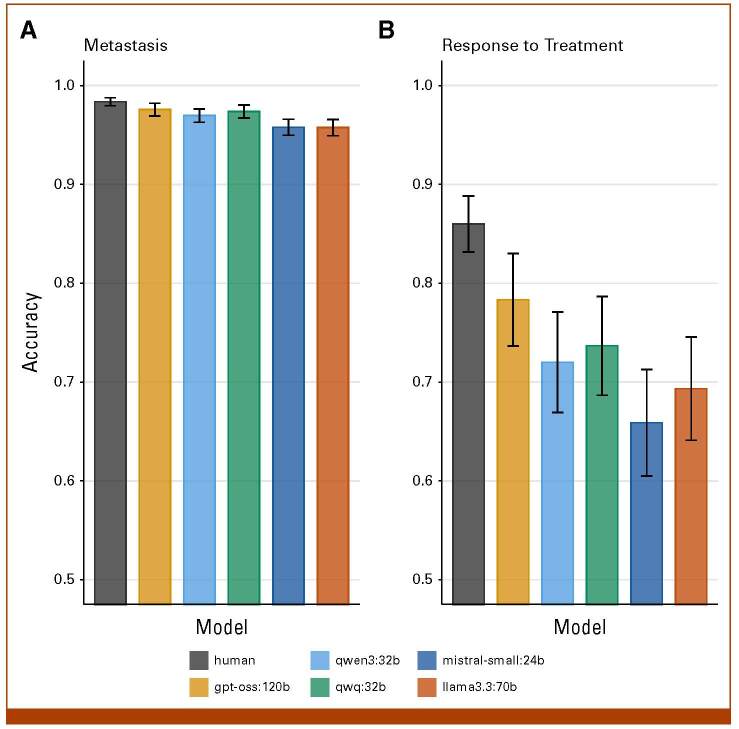
Plots show pooled accuracy and 95% CIs of humans and LLMs. (A) Binary classification of site-specific metastases (present *v* absent). (B) Multiclass classification of response to treatment (categories for CT/MRI: stable disease, response, remission, not applicable; for PET: stable disease, partial response, complete response, remission, not applicable). CT, computed tomography; LLMs, large language models; MRI, magnetic resonance imaging; PET, positron emission tomography.

Performance metrics were calculated exclusively on the test set (n = 300 reports). The ground truth for metastasis classification (presence *v* absence by site) contained 193 positive and 2,441 negative items. The human accuracy compared with ground truth was 98.4% (95% CI, 98.0 to 98.8). All LLMs met the prespecified noninferiority margin. gpt-oss:120b had the highest accuracy among LLMs (97.6%; 95% CI, 96.9 to 98.2), followed closely by the other models (range, 95.7%-97.4%; Table 2, Fig 1, Data Supplement, Fig S5). An analysis stratified by imaging modality (MRI *v* CT *v* PET-CT) showed similar results (Data Supplement, Fig S6).

**TABLE 2.** Pooled Accuracy and 95% CIs for Humans and LLMs, as Well as the Difference in Accuracy (LLM accuracy − human accuracy) With 90% CIs and Corresponding One-Sided Noninferiority *P* Values

Model	Accuracy, % (95% CI)	Difference From Human, Percentage Points (90% CI)	*P* Noninferiority
Metastasis			
Human	98.4 (98.0 to 98.8)	Reference	
gpt-oss:120b	97.6 (96.9 to 98.2)	–0.8 (–1.3 to –0.3)	<.001
qwen3:32b	97.0 (96.3 to 97.6)	–1.4 (–2.0 to –0.8)	<.001
qwq:32b	97.4 (96.7 to 98.0)	–1.0 (–1.5 to –0.5)	<.001
mistral-small:24b	95.8 (95.0 to 96.6)	–2.6 (–3.2 to –1.9)	<.001
llama3.3:70b	95.7 (94.9 to 96.6)	–2.6 (–3.3 to –1.9)	<.001
Response to treatment			
Human	86.0 (83.2 to 88.8)	Reference	
gpt-oss:120b	78.3 (73.7 to 83.0)	–7.7 (–11.6 to –3.8)	.869
qwen3:32b	72.0 (66.9 to 77.1)	–14.0 (–18.1 to –9.9)	>.999
qwq:32b	73.7 (68.7 to 78.7)	–12.3 (–16.3 to –8.4)	.999
mistral-small:24b	65.9 (60.5 to 71.3)	–20.1 (–24.5 to –15.8)	>.999
llama3.3:70b	69.3 (64.1 to 74.6)	–16.7 (–20.9 to –12.4)	>.999

NOTE. The upper panel reports binary classification of site-specific metastases (present *v* absent). The lower panel reports multiclass classification of response to treatment.

Abbreviation: LLMs, large language models.

**FIG 2.**
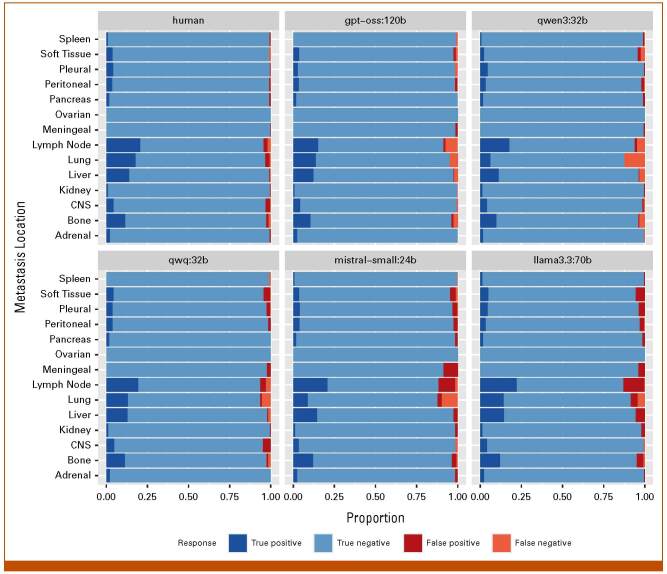
The plot shows the distribution of classification errors of metastasis status across anatomic sites, stratified by human coders and LLMs. Classification of findings in the lung and lymph nodes were the primary errors of humans. This was expected, given the more ambiguous language frequently used to describe findings in these sites. Interestingly, LLMs mainly struggled in the same areas. LLMs show different diagnostic profiles: for example, gpt-oss:120b and qwen3:32b appear to classify conservatively (higher threshold to classify a finding as metastasis), whereas llama3.3:70b and mistral-small:24b appear to classify findings as metastasis more liberally. LLMs, large language models.

In exploratory analyses, human coders achieved a PPV of 86.9% (95% CI, 83.1 to 90.8) and a NPV of 99.3% (95% CI, 99.1 to 99.6). LLM performance varied: gpt-oss:120b was conservative with a high PPV of 90.6% (95% CI, 86.0 to 95.1) and lower NPV (98.0%; 95% CI, 97.4 to 98.7). By contrast, llama3.3:70b had a low PPV (64.6%; 95% CI, 58.3 to 70.9) with an NPV of 99.4% (95% CI, 99.1 to 99.7; Data Supplement, Figs S7 and S8, and Table S2). Humans achieved an F1 score of 89.1% (95% CI, 86.3 to 91.5); qwq:32b achieved the highest F1 score among models at 82.7% (95% CI, 78.3 to 86.5), followed by gpt-oss:120b at 81.8% (95% CI, 76.9 to 86.3; Data Supplement, Table S2). Errors clustered in lung and lymph nodes (Fig 2), where gpt-oss:120b produced predominantly false negatives, whereas llama3.3:70b produced predominantly false positives.

### Response to Treatment

For assessment of treatment response, the human accuracy was 86.0% (95% CI, 83.2 to 88.8). No LLM achieved noninferiority. The best-performing model, gpt-oss:120b, achieved an accuracy of 78.3% (95% CI, 73.7 to 83.0; Table 2, Fig 1, Data Supplement, Fig S5). An analysis stratified by imaging modality (MRI *v* CT *v* PET-CT) showed similar results (Data Supplement, Fig S6).

When assessing treatment response by classification category in exploratory analyses, accuracy varied substantially (Data Supplement, Figs S9 and S10). LLMs underperformed humans within the *progression* or *stable disease* categories. For *progression*, humans were 96.7% accurate (95% CI, 93.1 to 99.3); the best performing models, mistral-small:24b and qwq:32b, achieved 77.4% (95% CI, 67.6 to 86.2), with other models ranging between 70.0% and 73.4%. For *stable disease*, the human accuracy was 74.1% (95% CI, 65.5 to 82.1); the best performing model, gpt-oss:120b, achieved 58.6% (95% CI, 46.5 to 70.0), whereas other models fell below 50% (Data Supplement, Table S3).

To assess the clinical utility, we simplified the task to a binary classification (progression *v* non-progression). In this setting, all LLMs achieved an NPV of >98% comparable with the human benchmark (99.0%; 95% CI, 97.9 to 99.8). However, poor specificity resulted in substantially lower model PPVs (range, 70.0%-77.5%) compared with humans (96.6%; 95% CI, 92.8% to 99.3; Data Supplement, Fig S11 and Table S4). Humans had a high F1 score of 96.1% (95% CI, 93.3 to 98.3). Among the models, mistral-small:24b achieved the highest F1 score at 86.6% (95% CI, 79.0 to 92.3), whereas gpt-oss:120b scored 82.1% (95% CI, 74.1 to 88.6; Data Supplement, Table S4).

### Secondary Outcomes

**FIG 3.**
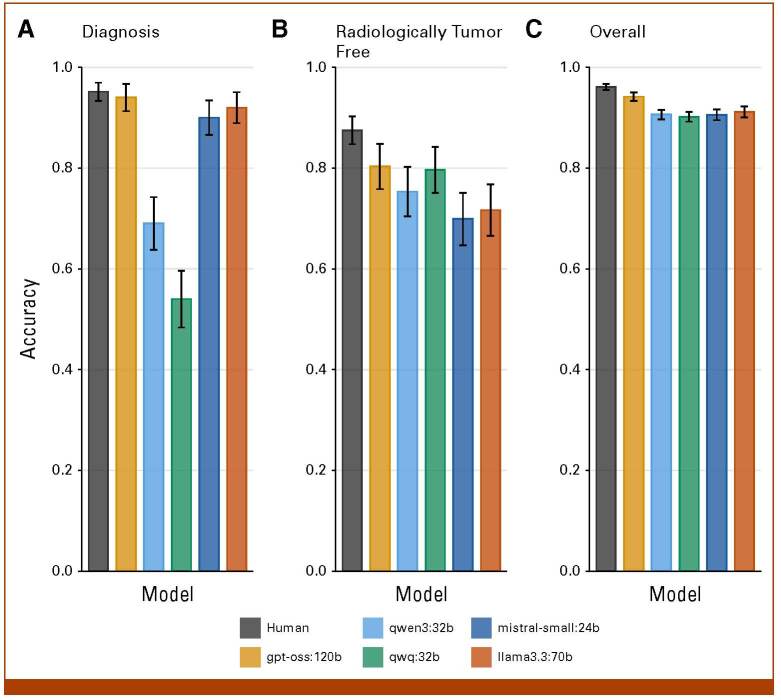
Plots show pooled accuracy and 95% CIs comparing humans and LLMs. (A) Multiclass classification of primary tumor diagnosis. (B) Binary classification of radiologic absence of tumor (yes *v* no). (C) Overall accuracy pooled across all primary (Fig 1) and secondary (A and B) end point tasks. LLMs, large language models.

For classification of the primary tumor diagnosis, the human accuracy was 95.2% (95% CI, 93.4 to 97.0). Only gpt-oss:120b was noninferior (94.0%; 95% CI, 91.3 to 96.7). Accuracy varied by tumor type, with models generally performing similarly to humans (Data Supplement, Fig S12), except for qwq:32b and qwen3:32b, which performed poorly. For the classification of radiologic tumor absence, the human accuracy was 87.5% (95% CI, 84.8 to 90.2). All LLMs were inferior to human classification. Across all tasks, only gpt-oss:120b was noninferior (Fig 3, Data Supplement, Table S5).

The mean human extraction time per report was 120s (range, 60-240s). LLM processing times per report were 11s (mistral-small:24b), 33s (llama3.3:70b), 45s (gpt-oss:120b), 47s (qwq:32b), and 63s (qwen3:32b).

### Sensitivity Analyses

A Bayesian sensitivity analysis yielded results consistent with the frequentist analysis (Data Supplement, Figs S13-S15). In a further sensitivity analysis to assess ground truth reliability, blinded senior oncologists reviewed all discrepancies between the ground truth and gpt-oss:120b for the primary outcomes. While the original ground truth was confirmed in most cases (metastasis: 48 of 64; response: 50 of 64), the changes were sufficient for gpt-oss:120b to achieve noninferiority for treatment response when evaluated against the revised ground truth (Data Supplement, Fig S16 and Table S6).

## DISCUSSION

In this study, we show that locally hosted LLMs can achieve human-level accuracy for explicit data extraction tasks in German radiology reports. Specifically, while all models identified metastatic sites with high accuracy, none achieved noninferiority for the more complex task of treatment response assessment. Similar results have been observed in different data sets in different languages.^2,28^

This performance decrease can likely be explained, in part, by data quality: the radiology reports often contained ambiguous language or omitted definitive conclusions, making ground truth establishment challenging. This ambiguity mirrors clinical reality, where determining whether imaging findings truly reflect a response is often time-consuming and requires interdisciplinary discussion. Human inter-rater agreement decreased for classification of treatment response and radiologic tumor absence. In addition, agreement between gpt-oss:120b and each human coder was similar to the agreement between human coders. This suggests that information required for a definitive classification was sometimes absent from the report itself. The apparent superiority of human classification of treatment response assessment compared with gpt-oss:120b might be in part attributable to the design: because the ground truth relied on human consensus, it inherently favored human performance. For example, if the both human coders made the same, incorrect classification, the resulting incorrect ground truth could falsely penalize LLMs. Our sensitivity analysis revealed that in approximately 20% of adjudicated discrepancies between the ground truth and gpt-oss:120b, the original ground truth was deemed incorrect. This further highlights that establishing a definitive gold standard is inherently difficult because of the complexity and ambiguity of medical reports. The bias in favor of human performance could be mitigated by having more than two independent human coders as this would make chance agreements on incorrect answers less likely. However, this would also require substantially more resources. In addition, integrating longitudinal data, such as previous imaging reports, clinical notes, and treatment changes, might improve accuracy but would significantly increase pipeline complexity.

We observed unexpected variability in model performance across tasks: qwen3:32b and qwq:32b performed robustly for metastasis classification but poorly for primary diagnosis classification, even after prompt optimization. This inconsistency suggests that performance cannot easily be extrapolated from one task to another; models should be benchmarked specifically for each intended use case before deployment.

We also found that implementation requires iterative prompt optimization and pipeline adaptation. We observed substantial performance gains by modifying our pipeline to allow models to generate a free-text reasoning step before outputting the structured JSON format, except for gpt-oss:120b, which performed best with a single prompt strategy.

Our study has several strengths. We adhered to rigorous clinical research standards, including preregistration of the study, extraction in duplicate, and adjudication by a senior oncologist. Furthermore, our conclusions are robust to multiplicity; when applying a Bonferroni correction for 10 comparisons (five models across two primary end points), the noninferiority (*P* < .001) of all models for metastasis status would remain unchanged.

Our study has limitations. The single-center, German-language setting limits generalizability to other languages and reporting styles. Furthermore, we were not able to compare the performance of LLMs with that of more established methods in natural language processing, such as Bidirectional Encoder Representation from Transformers, which have been successfully implemented for routine oncology data.^29^ This was unfeasible because we did not have the resources to annotate enough data for fine-tuning models for each classification task.^30^ The availability of only a single radiology report, without additional information or clinical context (eg, prior imaging or the patient's clinical status), represents an artificial scenario that might have adversely affected the performance of both human coders and LLMs. Finally, we restricted our evaluation to open-source, locally hosted models to prioritize data privacy. It is likely that larger commercial models would achieve higher accuracy.

Despite these limitations, LLMs could, when used carefully, improve research efficiency. For example, determination of progression-free survival, a critical end point in oncology, typically requires labor-intensive manual review of longitudinal imaging to identify the date of first progression. Automating the extraction of progression events or new metastases could significantly accelerate this process. While our findings suggest that current open-source solutions might not yet be robust enough for fully automated implementation, the rapid pace of advancement indicates that future models may increasingly become viable for these complex applications.

In conclusion, in this single-center study of German radiology reports, locally hosted LLMs were noninferior to human extraction for metastasis status but did not meet the prespecified noninferiority criterion for response-to-treatment classification. This may reflect not only model performance but also the difficulty in establishing a ground truth from ambiguous routine radiology reports. Successful integration of these tools into clinical research pipelines requires task-specific validation and careful consideration of model choice.

## Data Availability

We are unable to share the original clinical reports because they contain protected patient data; however, all analysis codes and codes used for prompting are available on GitHub.^20^
